# Additional funding mechanisms for Public Hospitals in Greece: the case of Chania Mental Health Hospital

**DOI:** 10.1186/1752-4458-4-27

**Published:** 2010-11-10

**Authors:** Anastasios Rentoumis, Nikolaos Mantzoufas, Gavriil Kouris, Christina Golna, Kyriakos Souliotis

**Affiliations:** 1 VIDAVO Health Telematics, 9th Thessaloniki - Thermi, THERMI 1 building, D8 Office, P.O. BOX 60521, 570 01 Thessaloniki, Greece; 2 Ministry of Economy and Finance, 2nd Floor, 8 Kageorgi - Servias Str., Syntagma Square, 101 80 Athens, Greece; 3 Department of Medicine, University of Crete, Heraklion, 71003, Greece; 4 Health Policy, Roche Hellas SA, 4 Alamanas Street, 15125, Maroussi, Greece; 5 Health Policy and Health Economics, Faculty of Social Sciences, University of Peloponnese, Corinth, 20100, Greece

## Abstract

**Objectives:**

To investigate whether the long term lease of public hospital owned land could be an additional financing mechanism for Greek public (mental) health hospitals.

**Methods:**

We performed a financial analysis of the official 2008 data of a case - study hospital (Mental Health Hospital of Chania). We used a capital budgeting approach to investigate whether value is created for the public hospital by engaging its assets in a project for the development of a private renal dialysis Unit.

**Results:**

The development of the private unit in hospital owned land is a good investment decision, as it generates high project Net Present Value and Internal Rate of Return. When the project commences generating operating cash flows, nearly €400.000 will be paid annually to the Mental Health Hospital of Chania as rent, thereby gradually decreasing the annual deficit of the hospital.

**Conclusions:**

Revenue generated from the long term lease of public hospital land is crucial to gradually eliminate hospital deficit. The Ministry of Health should encourage similar forms of Public Private Partnerships in order to ensure the sustainability of public (mental) hospitals.

## Introduction

Greek NHS Hospitals are financed through taxation via the state budget (primarily for infrastructure, salaries and equipment) and social security funds (for the reimbursement of services provided to their insureds). Current hospital debt for the 137 hospitals of the public sector amounts to €6 billion and the current fiscal crisis [1] puts even greater strain on public hospital sector financing, with forced budget cuts and increasingly irregular reimbursement by sickness funds [2]. This is expected to have an effect on hospital financial statements, with deficits increasing. It is clear that there is a need for a different financing paradigm for the Greek NHS hospitals.

More specifically, Public Mental Health Hospitals face a deficit of over €12 million in their annual budgets. Over the past 30 years, Public Mental Health Services in Greece have undergone a significant reform to modernize service provision according to WHO standards [3], with emphasis on de-institutionalization and community mental health care provision [4]. As a result, public mental health hospitals across the country gradually transferred chronic mental health patients to community housing units and closed down their respective hospital units. Nonetheless, according to Ministry of Health hospital accounts, the accounts receivable of mental health hospitals increased significantly, due to irregular payments by sickness funds and substantial delays of over two years in some cases. In tandem, the accounts payable to hospital providers have also increased (Table 1). As a result, cash inflows from operations to mental health hospitals have decreased over the last three years and this has had a dramatic impact on the quality and quantity of services provided to mental health users.

**Table 1:** Accounts Payable by Mental Health Hospitals to Providers and Accounts Receivable by Sickness Funds to Greek Mental Hospitals (1.1.2005 - 31.12.2008)

	Hospital	**Accounts Payable (in €) **[1]	**Accounts Receivable (in €) **[2]	**Difference **[2] - [1]**(in €)**
1	Child and Adolescent Mental Health Hospital of Athens	906.706,19	638.147,75	- 268.558,44

2	Dafni Mental Health Hospital	35.066.998,00	8.642.391,49	- 26.424.606,51

3	Dromokaitio Metal Health Hospital	295.251,95	7.801.007,73	7.505.755,78

4	Katerini Mental Health Hospital	264.983,80	1.592.448,37	1.327.464,57

5	Corfu Mental Health Hospital	1.052.083,20	2.032.132,58	980.049,38

6	Tripoli Mental Health Hospital	518.416,09	2.256.399,88	1.737.983,79

7	Chania Mental Health Hospital	1.036.665,91	1.857.276,17	820.610,26

8	Thessaloniki Mental Health Hospital	7.562.362,37	3.563.502,59	- 3.998.859,78

9	Leros Mental Health Hospital	1.127.976,06	6.547.900,95	5.419.924,89

**SUM**		**47.831.443,57**	**34.931.207,51**	**- 12.900.236,06**

Despite the dire fiscal situation of most public hospitals, very little has been done at the legislative level to promote alternative funding solutions such as the optimization of the use of publicly owned land and property. The Hellenic Public Real Estate Corporation has repeatedly suggested that the book value of fixed assets owned by the public sector exceeds €300 billion. There is, however, no information available from the Ministry of Health concerning the book value of fixed assets of Greek hospitals. In addition to that, only 80 out of 137 public hospitals have implemented the double entry method for recording financial transactions and publish annual financial statements.

This paper examines whether the long term lease of hospital property to investors to develop and operate a private Renal Dialysis Unit in the premises of the mental health hospital of Chania is a sustainable and attractive additional funding mechanism for the hospital. Since fully deinstitutionalizing its services by closing down all hospital units and transforming them into community mental health units, the mental health hospital of Chania, in Crete, was faced with an accumulated budget deficit and very limited cash inflows. Hospital Management developed an action plan for PPP/PF Initiatives that aimed at raising additional funds. Prominent in the action plan is the prospect of leasing out hospital property to establish a private Renal Dialysis Unit, for which current and future expected demand in the prefecture is high [5].

Conclusions may be transferrable to other public hospitals (non specialized) and of significance to health policy decision makers to assess whether additional cash flow for significantly strained health units around Greece could be realized through a coordinated PFI/PPP programme.

## Methodology

We performed a financial Analysis of 2008 hospital data. The hospital balance sheet and income statement were prepared according to the Greek General Chart of Accounts [6]. Financial data were collected and analyzed according to the double - entry accounting method, but financial analysis was not performed according to the International Financial Reporting Standards since financial statements would be prepared for only the second consecutive year in 2009. A feasibility study was carried out in mid 2008 to investigate the potential for value creation through a mechanism of real estate management that will maximize returns to the hospital. The following steps were taken to assess the viability of real estate investment projects in the premises of the mental health hospital, which remained closed since late 2006 when deinstitutionalization was concluded:

**1. **Recording of Land and Buildings (fixed assets) using the replacement cost principle and depreciation accords to the straight - line depreciation method. Fixed assets and depreciation expenses were recorded in the 2008 Balance Sheet.

**2. **Market research analysis to investigate the demand for real estate investments in the premises of the hospital. This included on the spot visits from potential investors and a non - binding, informal session of Request for Proposals from interested investors.

**3. **Feasibility study, assessing the financial viability of investment proposals for real estate development. In addition, multiple discussions took place between hospital management, project consultants (GRANT THORNTON S.A. GREECE) and local municipalities for the fair treatment and mutual support in the realization of the project.

**4. **Recording of value creation indicators for projects (Net Present Value and Internal Rate of Return). For the project with +ve Net Present Value (NPV) and Internal Rate of Return (IRR) larger than the Weighted Average Cost of Capital (WACC), a long term rent was negotiated with the potential investor. Informal PFI/PPP proposals from public institutions for long term rent of land were also presented to the management board of the hospital. The results of the process were presented to the Ministry of Health and Social Solidarity for decision making.

## Results

According to the 2008 Balance Sheet, at December 31st, 2008, land and buildings, minus accumulated depreciation, represented 59% of total assets whereas accounts receivable represented 20% of total assets and cash only 7% of total assets. At January 1st 2008, the figures were 64%, 15% and 13% respectively. As far as liabilities were concerned, at December 31st, 2008, accounts payable represented 21% of total liabilities whereas at January 1st 2008, the relevant figure was 10%. It appears that over time accounts payable increase, cash equivalents decrease and accounts payable increase. At the same time, the value of property (land and buildings) decreases due to depreciation. According to the Income Statement for 2008 net income amounted to €1.682.960, whereas loss amounted to €1.245.495. According to the fiscal reality in the Greek hospitals, this is a small deficit, but is expected to increase over time given the financial crisis the Greek economy faces.

A feasibility study was performed to assess whether it would be viable to construct a, private renal dialysis unit within the premises of the hospital and estimate cash flows from the operation of this unit over a substantial time period (30 years). The Private Renal Dialysis Unit would operate throughout the year serving local renal dialysis patients as well as tourists in need of renal dialysis, with a capacity of 30 renal dialysis beds. Such a unit would have a surface area of 2.500 m^2^. Land required for construction would have a surface area of about 8,000 m^2^. The investor would pay long term rent to the hospital, in monthly installments for the use of land.

For 2010, the Cost of Investment for the unit was estimated to be €4.16 m and is presented in table 2. The financial structure of the investment is presented in table 3. The expected revenue from the operation of the unit is presented in table 4.

**Table 2:** Cost of Investment of the Renal Dialysis Unit

COST OF INVESTMENT (in €)	
Construction Cost	3.000.000

Cost of Surroundings	200.000

Cost of Equipment	960.000

**SUM**	**4.160.000**

**Table 3:** Financial Structure of the Investment Cost

INVESTMENT BUDGET	4.160.000 €	
	Bank Loans = 40%, Investors Equity = 60%	

Construction Period	2 years	

Year 1	50%	2.080.000 €

Year 2	50%	2.080.000 €

**SUM**		4.160.000 €

Investors Equity		

Year 1	60%	1.248.000 €

Year 2	60%	1.248.000 €

Bank Loans		

Year 1	40%	832.000 €

Year 2	40%	832.000 €

**Table 4:** Income Analysis (2010 prices, 3.5% increase per year)

REVENUE ANALYSIS				
As % of sessions	56%	44%		100%

Number of sessions	**10.022**	**7.795**		**17.817**

Structure according to the type of dialysis method		***Hemodialysis***	***Variations of Hemodialysis***	
	
	100%	60%	40%	100%

Number of Sessions	**10.022**	**4.677**	**3.118**	**17.817**

Price of a Session	250 €	121 €	178 €	

Annual Revenue	**2.505.488 **€	**565.921 **€	**555.008 **€	**3.626.416 **€

As far as operating costs are concerned, these sum up to about €2.4 m (2010 prices), of which €187.200 are fixed costs, €723.726 marginal costs, €910.000 personnel costs and €400.000 annual rent paid to the hospital and to the Ministry of Health. The Profit and Loss Account shows a steady increase in Earnings after Tax over the 30 year period of the rent process (table 5).

**Table 5:** Profit and Loss Account for the Renal Dialysis Unit, 2010 - 2014

	2010	2011	2012	2013	2014
Net Sales (Revenues)	3.626.416	3.753.340	3.884.707	4.020.672	4.161.395

Cost of Services Sold	2.588.078	2.678.661	2.772.414	2.869.449	2.969.880

**GROSS PROFIT**	**1.038.337**	**1.074.679**	**1.112.293**	**1.151.223**	**1.191.516**

Interest Expenses	113.332	98.709	84.085	69.462	54.838

**SUM**	**925.005**	**975.970**	**1.028.208**	**1.081.761**	**1.136.678**

Depreciation	226.667	226.667	226.667	226.667	227.475

**Earnings before Tax**	**698.338**	**749.304**	**801.541**	**855.095**	**909.202**

Tax	174.585	187.326	200.385	213.774	227.301

**Earnings after Tax**	**523.754**	**561.978**	**601.156**	**641.321**	**681.902**

**Dividends**	**523.754**	**561.978**	**601.156**	**641.321**	**681.902**

The Weighted Average Cost of Capital in March 2008 was estimated to be 12.38%. The Net Operating Cash Flows for the period between 2010 and 2040 give a Net Present Value of €2.8 m and a project IRR of 23%, about 11% higher than Weighted Average Cost of Capital.

From both those indicators of value creation, the investment is sustainable and highly recommended for potential investors.

On the hospital side, the feasibility study indicated that the hospital could realize revenue from the long term lease to private and public investors. Project financing would be either through investors capital and bank loans (in the case of private investors) or through the PFI scheme (in the case of public sector investors). According to the PFI methodology [7], the private sector undertakes the risk of designing, financing and constructing public infrastructure and the public sector undertakes the risk of meeting demand. In this model, the public sector pays the private sector by regular installments. This paper considers only cash flows generated by private investment, given the current fiscal difficulties confronting public investment.

## Discussion

In times of fiscal difficulty not only for the Health Sector but for the Greek Public Sector as a whole, mental health reforms currently underway are endangered by lack of funding. Mental Health Hospitals face immense delays in being paid accounts receivable by sickness funds and practically lack any alternative funding. This undermines not only the efficiency of the mental health sector but also the quality of services.

Given the current hospital funding system, to ensure the long term financial sustainability of mental health hospitals, it is important to design a central plan for the development of public hospital property and implement Public Private Partnerships so that annual cash inflows could be realized.

As results from the analysis performed at Chania Mental Health Hospital indicate, one strategy for hospital property development is the long term lease of land to potential investors that would develop value creating investments, such as the development of a Renal Dialysis Unit for locals and tourists.

In the case study hospital, the strategy for asset development and value creation was the long - term lease of hospital property, under the contractual obligation of the successful bidder to build new and/or redevelop existing infrastructure and operate it for the duration of the lease period. No option for acquisition of the assets was given, at least at this stage, to the investor.

Data indicate that the investment has a high NPV and IRR and creates substantial value for the investor. Long term lease of property also generates significant revenue for the hospital that is expected to aid the hospital in the long run to decrease its deficit, thereby enhancing efficiency and quality of services offered.

This paper is a first attempt to describe and quantify the advantages of alternative uses of public property in mental health care in Greece. As such, this paper is faced with the limitation of lack of Greek data as well as other literature references. Also lacking are evidence on cost effectiveness issues regarding the closure of psychiatric hospitals and the development of community mental health services for various populations of service users, thus limiting the construction of a full econometric model. In addition international comparisons are not appropriate, as in most European countries deinstitutionalization of mental health services and consequently change of use of public mental hospitals was discussed at least twenty years ago [8-10]. Although there is plenty of literature on the shift from hospital mental health care to community mental health care provision, there is very little information on capital asset management of former mental health hospitals. Nonetheless and in certain cases, speeding up of processes for selling or leasing vacant psychiatric hospital sites in order to release resources valuable for mental health reform was suggested [10]^.^

## Conclusion

Given the lack of a centralised plan on the optimization of hospital owned land and property, it is imperative that the Ministry of Health proceeds as soon as possible to similar sustainability studies for hospital property development. In addition, when these studies are concluded, Public Procurement Processes should be carried out to attract interested investors that are willing to lease land and buildings owned by public (mental) health hospitals on a long term basis.

Authors strongly recommend the systematic and sustainable collection of relevant mental hospital data as well as community mental care data to better facilitate an in-depth, comparative analysis of key metrics in a life-time horizon.

## Competing interests

The authors declare that they have no competing interests.

## Authors' contributions

AR collected and analyzed hospital specific data as part of his hospital management position. NM provided input on PPP/PFI legal and operational framework in Greece. GK reviewed international available literature. CG discussed findings. KS analyzed the results and overviewed the whole paper. All authors read and approved the final manuscript.
